# Comparative Toxicological Study between Exposed and Non-Exposed Farmers to Organophosphorus Pesticides 

**DOI:** 10.22074/cellj.2016.3991

**Published:** 2016-04-04

**Authors:** Fariba Taghavian, Gholamhassan Vaezi, Mohammad Abdollahi, Ali Akbar Malekirad

**Affiliations:** 1 Department of Biology, Damghan Branch, Islamic Azad University, Semnan, Iran; 2 Faculty of Pharmacy and Pharmaceutical Sciences Research Center, Tehran University of Medical Science, Tehran, Iran; 3 Department of Biology, Payame Noor University, Tehran, Iran

**Keywords:** Genotoxicity, Organophosphorus Pesticides, Acetylcholinesterase, Interleukins, Farmers

## Abstract

**Objective:**

The purpose of this work was to compare DNA damage, acetylcholinesterase (AChE) activity, inflammatory markers and clinical symptoms in farmers exposed to organophosphorus pesticides to individuals that had no pesticide exposure.

**Materials and Methods:**

We conducted a cross-sectional survey with a total of 134 people. The subject group consisted of 67 farmers who were exposed to organophosphorus pesticides. The control group consisted of 67 people without any contact with pesticides matched with the subject group in terms of age, gender, and didactics. Oxidative DNA damage, the activities of AChE, interleukin-6 (IL6), IL10 and C-reactive protein (CRP) in serum were measured and clinical examinations conducted in order to register all clinical signs.

**Results:**

Compared with the control group, substantial gains were observed in the farmers’ levels of oxidative DNA damage, IL10 and CRP. There was significantly less AChE activity in farmers exposed to organophosphorus pesticides. The levels of IL6 in both groups did not significantly differ.

**Conclusion:**

The outcomes show that exposure to organophosphorus pesticides may cause DNA oxidative damage, inhibit AChE activity and increase the serum levels of inflammatory markers. Using biological materials instead of chemical pesticides and encouraging the use of safety equipment by farmers are some solutions to the adverse effects of exposure to organophosphorous pesticides.

## Introduction

Nowaday, with the growth of farming and widespread use of chemicals and organophosphorus pesticides, major problems may occur in the nervous, endocrine, reproductive and immune systems of people in addition to numerous chronic diseases such as cancer (1). In this case, the human detoxification system plays a critical function in reducing the harmful effects of pesticides. Toxin levels that increase above the detoxification system’s capacity cause worsening health conditions. The intake of antioxidants such as vitamins C and E, as well as organic crops may play an important role in reducing the harmful effects of pesticides through increasing blood antioxidant capacity (2, 3). Also, the evolution of the pesticide industry and the indiscriminate usage of pesticides cause adverse effects on human health, food, and environment. Assessing oxidative stress parameters in plasma is an important means to detect oxidative stress induced by organophosphorus pesticides (3). Organophosphorus pesticides may inhibit acetylcholinesterase (AChE) activity as one of the markers of oxidative stress in workers exposed to these substances (4). In addition to AChE inhibition, 8-hydroxy- 2-deoxy guanosine (8-OHdG) level is an oxidative DNA damage indicator to determine the amount of oxidative stress caused by organophosphorus pesticides (5, 6). Cholinergic syndrome is the most important neurotoxic disorder that results from vulnerability to these pesticides (7). Organophosphorus pesticides, by affecting liver cells, increase the release of glucose through glycogenolysis and gluconeogenesis processes and can be seen as a diabetes risk factor (8, 9). Hence, the genetic risk of pesticide exposure is dangerous for humans. Pesticides cause DNA damage in workers involved in producing these compounds. However, these damages are much less in those who observe safety precautions (10). Persistent organic pollutants (POPs) are chemicals that resist biodegradation and are toxic, and are mostly discharged into the environment by industrial activities. Various animal studies have shown the effects of POPs in different inflammatory markers (11). C-reactive protein (CRP), a plasma protein marker of inflammation in humans, belongs to the pentraxin group of molecules was discovered in 1930. Its gene is located on chromosome 1. CRP is a member of the defense molecules known as acute phase proteins (12). This molecule has been used over a number of years as an inflammatory marker in infectious and inflammatory diseases (13).

The effects of acute exposure to a variety of pesticides on human health are well known. However, the effects of long term exposure to low-dose pesticides on various aspects of human health, including genotoxicity and inflammatory markers, are not clearly defined. Despite the fact that some studies show the effects of pesticides on induced immunity, there is little evidence of severe immune disorders in humans (14). It seems that although the exposure to pesticides can alter immune function, the emergence of immune disorders is related to the dose and duration of exposure to pesticides (15). The current study aims to investigate the effects of chronic exposure to low doses of organophosphate pesticides on genotoxicity and inflammatory markers in farmers. Farmers are exposed to different types of pesticide during the spraying season. Since pesticides can endanger the physical and mental health of people, they impose tremendous expense on individuals and society. Thus, considering the extensive cognitive and physical effects of pesticides on health and the enormous economic costs imposed on society for treating these disorders, the present work has intended to compare DNA damage, AChE activity and the inflammatory factors in farmers exposed to organophosphorus pesticides to a control group of unexposed individuals living in a village in the vicinity of Arak, Iran.

## Materials and Methods

This cross-sectional survey enrolled 134 people. The study group consisted of 67 farmers who resided in a village near Arak in the Central province of Iran who were exposed to organophosphorus pesticides for at least 5 years.

The control group consisted of 67 subjects who were not farmers and lived in a nearby village. Control group participants had no exposure to pesticides and were matched with the study group in terms of age, gender, and education. All subjects had expressed their written informed consent before they entered the experiment. The study was conducted in complete accordance with the National Code of Ethics and approved by the Ethics Committee of the Islamic Azad University of Damghan Branch.

Interviews with the farmers determined that they used no proper protective equipment such as aprons, boots, filtered masks, or goggles. All subjects in this study underwent clinical examinations to determine the presence of any symptoms of chronic conditions such as high blood pressure, heart problem, cancer, thyroid disorder, asthma, diabetes and anemia. The data concerning job history, smoking, drug consumption, vitamins and antioxidant supplements were obtained through a questionnaire. After clinical examinations, farmers with chronic diseases, alcohol and antioxidant use, or those under pharmacological therapy were excluded.

A total of 5 ml of venous blood was taken from each person in both the subject and control groups. After serum separation by centrifugation, oxidative DNA damage was studied by measuring 8-OHdG (ng/ml) as a bio-marker of oxidative stress and the product of the DNA repair enzyme with an enzyme-linked immunosorbent assay (ELISA) kit (Cayman Chemical Co., USA). For the measurement of cholinesterase activity in plasma, a total of 3 ml from the incubation solution of a potassium phosphate buffer (75 mmol/L, pH=7.9) and 5,5'-dithiobis-(2-nitrobenzoic acid) (DTNB) reagent (0.25 mmol/L) were added to 10 μl of the serum samples. The activity of the enzyme was induced by the addition of 10 μl of acetylthiocholine iodide (3 mmol/L). Absorption changes were measured with a spectrophotometer at 412 nm. We used the extinction coefficient of 5-thio-2-nitrobenzoic acid (TNB) to measure AChE activity (16).

To assess the level of CRP (ng/ml), we used the highly sensitive ELISA [highly sensitive-CRP, (hs-CRP)] and a kit by Bender Med Systems Inc., Austria. The concentrations (pg/ml) of interleukin-6 (IL6) and IL10 in serum samples were evaluated by ELISA and the kits from the above mentioned company. ELISA test procedures were carried out according to the protocol proposed by each kit.

To assess the impact of gender, the levels of 8-OHdG, serum acetylcholine esterase activity and inflammatory factors in male farmers exposed to pesticides were compared with male farmers with no pesticide exposure. The same analysis was performed for females.

### Statistical analysis

SPSS version 18 (IBM Inc., USA) was used to analyze the data. For this purpose, statistical analysis (independent samples t test) and descriptive statistics (mean ± SD) were performed. The P values lower than 0.05 were considered statistically significant. Pearson’s correlation coefficient was used to determine relationships between variables.

## Results

The total study population consisted of 134 people, 67 subjects and 67 controls, of whom 52.2% were male and 47.8% were female. The subjects were between 16 to 86 years of age. The majority (98.2%) had less than a diploma.

According to Table 1, there was a significantly greater increase in the level of 8-OH2dG in the subject group (30.41 ± 15.81 ng/ml) compared to the control group (26.04 ± 7.22 ng/ml, P<0.05). Serum AChE activity significantly decreased in the subject group (5098.82 ± 558.81 U/l) compared to the control group (5397.26 ± 574.05 U/l, P<0.05. (There was no significant difference in IL6 levels between the subject (1.80 ± 0.61 pg/ml) and control (1.62 ± 0.49 pg/ml) groups (P>0.05). The level of IL10 in the subjects was 1.98 ± 0.62 pg/ml and 1.38 ± 0.77 pg/ml for the control group. This was a significant increase in the subjects compared with the controls (P<0.05). CRP level significantly increased in the subject group (2.41 ± 0.69 ng/ml) compared to the control group (2.17 ± 0.60 ng/ml, P<0.05, Table 1).

**Table 1:** Comparison of the levels of 8-hydroxy-2-deoxy guanosine (8-OH2dG), serum acetylcholinesterase activity (AChE) and inflammatory markers in farmers exposed to organophosphorus pesticides to unexposed controls


	Subjects (n=67)	Controls (n=67)	P value

8-OH2dG (ng/ml)	30.41 ± 15.81	26.04 ± 7.22	0.04
AChE (U/l)	5098.82 ± 558.81	5397.26 ± 574.05	0.003
IL6 (pg/ml)	1.80 ± 0.61	1.62 ± 0.49	0.06
IL10 (pg/ml)	1.98 ± 0.62	1.38 ± 0.77	0.001
CRP (ng/ml)	2.41 ± 0.69	2.17 ± 0.60	0.03


IL; Interleukin and CRP; C-reactive protein.

According to Table 2, in male farmers exposed to pesticide, the level of 8-OH2dG was 34.61 ± 17.78 ng/ml compared to 24.52 ± 5.14 ng/ml in control group males, which was a significant increase (P<0.05). Serum AChE activity significantly decreased in male subjects (5137.17 ± 560.72 U/l) and compared to the control male (5441.31 ± 536.07 U/l, P<0.05.( IL6 levels in male subjects was 1.60 ± 0.50 pg/ml and in male controls it was 1.62 ± 0.49 pg/ml which showed no significant difference (P>0.05). The level of IL10 in male subjects was 1.91 ± 0.52 pg/ml which significantly increased compared to unexposed control males (1.19 ± 0.76 pg/ml, P<0.05). CRP level in exposed male farmers was 1.41 ± 0.41 ng/ml and 1.48 ± 0.39 ng/ml in males without exposure, which did not significantly differ (P>0.05, Table 2).

According to Table 3 the level of 8-OH2dG was 25.81 ± 11.99 ng/ml in subject females compared to 27.71 ± 8.74 ng/ml in control females, which showed no significant difference (P>0.05). Serum AChE activity in subject females was 5056.89 ± 562.60 U/l and 5349.09 ± 617.91 U/l in female unexposed control farmers, which showed no significant difference (P>0/05). There was a significant increase in IL6 levels in subject females (2.02 ± 0.65 pg/ml) compared to control females (1.63 ± 0.50 pg/ml, P<0.05). IL10 levels also significantly increased in female subjects (2.05 ± 0.71 pg/ml) compared to female controls (1.58 ± 0.74 pg/ml, P<0.05). CRP levels in exposed female farmers was 1.31 ± 0.42 ng/ml and 1.38 ± 0.39 ng/ml in female controls which showed no significant difference (P>0.05, Table 3).

**Table 2:** A comparison of the levels of 8-hydroxy-2-deoxy guanosine (8-OH2dG), serum acetylcholinesterase (AchE) activity and inflammatory factors in male farmers exposed to organophorphorus pesticides with unexposed male farmers


	Exposed male farmers (n=35)	Unexposed male farmers (n=32)	P value

8-OH2dG (ng/ml)	34.61 ± 17.78	24.52 ± 5.14	0.003
AChE (U/l)	5137.17 ± 560.72	5441.31 ± 536.07	0.02
IL6 (pg/ml)	1.60 ± 0.50	1.62 ± 0.49	0.87
IL10 (pg/ml)	1.91 ± 0.52	1.19 ± 0.76	0.001
CRP (ng/ml)	1.41 ± 0.41	1.48 ± 0.39	0.42


IL; Interleukin and CRP; C-reactive protein.

**Table 3:** A comparison of the levels of 8-hydroxy-2-deoxy guanosine (8-OH2dG), serum acetylcholinesterase (AchE) activity and inflammatory factors in female farmers exposed to organophosphorus pesticides with unexposed female farmers


	Exposed female farmers (n=32)	Unexposed female farmers (n=32)	P value

8-OH2dG (ng/ml)	25.81 ± 11.99	27.71 ± 8.74	0.47
AChE (U/l)	5056.89 ± 562.60	5349.09 ± 617.91	0.052
IL6 (pg/ml)	2.02 ± 0.65	1.63 ± 0.50	0.009
IL10 (pg/ml)	2.05 ± 0.71	1.58 ± 0.74	0.01
CRP (ng/ml )	1.31 ± 0.42	1.38 ± 0.39	0.48


IL; Interleukin and CRP; C-reactive protein.

According to Pearson correlation analysis, no significant correlation existed between the levels of 8-OH2dG and serum AChE activity to inflammatory factors (Table 4, P>0.05), nor were there any significant correlations observed between age and the levels of 8-OH2dG, AChE activity, IL6, IL10 and CRP (P>0.05, Table 5).

**Table 4:** The correlations between 8-hydroxy-2-desoxyguanosine (8-OH2dG) levels and serum acetylcholinesterase (AChE) activity with inflammatory factors


	IL6 (pg/ml)	IL10 (pg/ml)	CRP (ng/ml)

8-OH2dG (ng/ml)	-0.20	-0.15	-0.15
AChE (U/l)	0.09	0.79	0.42


IL; Interleukin and CRP; C-reactive protein.

**Table 5:** The correlations between age to 8-hydroxy-2-deoxy guanosine (8-OH2dG) levels, acetylcholinesterase (AchE) activity and inflammatory factors


	8-OH2dG (ng/ml)	AChE (U/l)	IL6 (pg/ml)	IL10 (pg/ml)	CRP (ng/ml)

Age (Y)	0.006	-0.05	-0.08	-0.08	0.14


IL; Interleukin-6 and CRP; C-reactive protein.

## Discussion

According to the results of this study, we observed a significant decrease in AChE activity in farmers exposed to organophosphorus pesticides compared to the control group. On the other hand, the extent of DNA damage, IL10 and CRP significantly increased compared to the controls. These results indicated that the farmers’ exposure to organophosphorus pesticides could produce free radicals, genomes and tissue damage. Farmers, when interviewed, stated that they did not use proper protective equipment during farming.

There was a substantial decrease in AChE activity in the blood serum of farmers exposed to organophosphorus pesticides compared with the control group, which reflected the high degree of pesticides absorbed. Earlier studies described the inhibition of AChE activity in farmworkers who sprayed pesticides (17). Farmers exposed to organophosphorus pesticides had a 30% reduction in blood AChE levels occurred compared with the control group (6). Manu et al. (18) reported a low activity of serum cholinesterase to be an indispensable component in the high rate of deaths due to acute organophosphorus poisoning.

Shadnia et al. (19) reported no substantial differences in AChE levels between farmers and controls. Cholinesterase levels in their study were consistent with those reported by Rastogi et al. (17) and Atherton et al. (6), however have contradicted results reported by Shadnia et al. (19). This contradiction may be attributed to the differences in pesticide dosage or duration of exposure. The main mechanism of action of organophosphorus compounds is to inhibit carboxylic ester hydrolase, particularly AChE. Organophosphorus compounds inhibit AChE using phosphorylation of the serine hydroxyl group located at the active site of the enzyme (20).

These compounds inhibit the actions of both cholinesterases, chain enzymes in red blood cell (RBC) and serum AChE-resulting in the cholinergic features of organophosphorus toxicity. RBC AChE activity, which is reduced at a lower rate than the serum AChE activity, is a measure of chronic exposure to organophosphates. Therefore, in organophosphorus poisoning, the activity of serum ChE reduces before the activity of RBCs (RBC-ChE) (21).

In the present study, we observed no significant relationship between age and serum AChE activity in the farmers. In the study done by Zheng et al. (22) age had no statistical significance in cholinesterase activity. Fairbrother et al. (23) reported that age significantly affected ChE activity. The present study supported results reported by Zheng et al. (22), on the other hand, it was inconsistent with the results reported by Fairbrother et al. (23). Young people have stated they are less willing to use health and safety equipment such as masks, boots, apron, or perform hand washing, and showering. Increased use of these measures will lead to less absorption of organophosphates. The absorption of organophosphates is associated with AChE activity which may justify the obtained results.

In this study, serum AChE activity in the exposed subject group significantly decreased compared to control, unexposed males. Serum AChE activity in exposed and unexposed female farmers did not significantly differ.

Sidell and Kaminskis (24) reported higher plasma cholinesterase activities in adult men during their first six decades of life compared to women; however, there was no difference between the two sexes after the age of 60. In a study by Fairbrother et al. (23) the plasma cholinesterase activity was affected by sex steroid concentrations and phases of the menstrual cycle. Thus, a significant positive correlation was found between serum cholinesterase activity and progesterone level. The present study agreed with the results by Zheng (22); on the other hand, the current study results were inconsistent with the results obtained by Fairbrother et al. (23). Physiological and genetic factors, lifestyle, nutrition and diet could make a difference in the levels of 8-OH2dG, acetylcholine esterase serum and inflammatory factors in both sexes.

Serum levels of 8-OH2dG in individuals exposed to organophosphorus pesticides significantly increased compared with the control group. Increased 8-OH2dG, as a biomarker of DNA damage, have indicated an increased risk of mutagenesis in these subjects (25, 26). Nuclear and mitochondrial DNA is biologically one of the primary targets of oxidative attack for free radicals. Among the purine and pyrimidine bases, guanine oxidation is more prone to oxidation in such a manner that as the result of a hydroxyl radical attack on the eighth position of guanine molecules results in production of 8-OHdG (27).

The levels of 8-OHdG and other modified bases which are separated from DNA and excreted from cells show a dynamic equilibrium between the rate of oxidative DNA damage and its repair. Thus, the level of oxidized bases changes not only as the result of oxidative damage repair but also as the result of fluctuations in the repair rate (28). Numerous studies have considered 8-OHdG to be a bio-marker of cellular oxidative stress and DNA repair product (29). The mutagenic property of 8-OHdG is known (30). Atherton et al. (6) have reported increased DNA damage in farmers exposed to organophosphorus compounds compared with the control group. In another study, exposure to organophosphorus compounds in young children was linked to increased levels of urinary 8-OHdG (31). In study by Muniz et al. (32) urinary 8-OHdG levels in farmers exposed to organophosphorus pesticides were higher than the control group. Organophosphorus pesticides could cause oxidative DNA damage in farmers, therefore the increased serum levels of 8-OH2dG in the present study were consistent with other surveys. CRP level in people exposed to organophosphorus pesticides was higher compared to the control group.

These results have strengthened the hypothesis that organophosphorus pesticides can affect the immune system and disrupt IL. In surveys conducted by Patil et al. (33), a substantial increase in serum CRP of vineyard spraying workers has been noted compared to controls. According to Tsai et al. (34) CRP levels strongly correlated with the level of severity of organophosphorus poisoning, such that the CRP level was important as an acute phase reactant in severe organophosphorus poisoning.

The outcomes of the present work were consistent with results obtained by Patil et al. (33) and Tsai et al. (34). The CRP molecule is an acute phase serum protein. In case of inflammation or infection this molecule is increasingly made by the liver. Increased levels of CRP are related to infection, age, body volume index, smoking, drug and dietary habits (35).

IL6 levels in both groups did not significantly differ. Kumar et al. (11) have stated that no significant relationship exists between the levels of POPs and IL6 which is consistent with the findings of this study.

Although exposure to pesticides can alter immune function, the emergence of immune disorders are dependent on the dose and duration of exposure to pesticides (15). The studies on the effects of pesticides on human immune system have many limitations, such as a shortage of data on exposure levels, heterogeneity of the methods employed, difficulty in providing a prognostic significance to the slight changes often observed and the interpretation of reported findings (36).

In this study, the exposure to organophosphorus pesticides led to a significantly increased IL10 serum level in farmers. Alluwaimi and Hussein (37) reported that the immunotoxicity caused by organophosphorus pesticides such as diazinon led to increased levels of IL10 in mice. The outcomes of this study were consistent with those reported by Alluwaimi and Hussein which indicated the effects of organophosphorus pesticides on the immune system. In studies by Neishabouri et al. (38) the dose dependent induction of diazinon affected the actions of both humoral and cellular immunities. In another study, malathion was used as an organophosphorus pesticide to induce increased activity of liver enzymes in plasma and pro-inflammatory cytokines. Thus, malathion has been shown to induce liver inflammation and hepatotoxicity (39). Organophosphorus compounds have toxic effects on immune systems and immune functions of vertebrates, invertebrates and fish. Immunotoxicity may be directed via inhibition of serine hydrolases or esterases in components of the immune system through oxidative damage to immune organs or by modulation of signal transduction pathways that control immune function. Indirect effects involve modulating of the nervous system or chronic effects of altered metabolism/nutrition on the immune system. Immunotoxicities are varied and include pathology of immune organs, and decreased humoral and/or cell mediated immunity. Modified non-specific resistance, reduced host resistance, hypersensitivity and autoimmunity are among the symptoms of immunotoxicity (40).

According to Pearson correlation analysis, no significant relation existed between 8-OHdG and AChE activity to inflammatory factors.

In a study performed by Kumar et al. (11), there was no significant relationship observed between POPs and inflammatory factors such as IL6 and CRP. Karanlık compared experimental data from two groups of patients who either died or were discharged with organophosphate poisoning. There was no statistically significant differences between the levels of IL6 in these patients (41). The findings in present study were consistent with those by Karanlık et al. (41) and Kumar et al. (11). Despite the large number of experimental data which have shown that induced immune safety is affected by pesticides, there is little or no evidence of severe immune disorders in humans caused by pesticides (14). Although exposure to pesticides can alter immune function, immune disorders are related to dose and duration of exposure to pesticides (15) and the low activity of cholinesterase is an essential factor for mortality rates by organophosphate poisoning (41).

## Conclusion

Exposure to organophosphorus pesticides can cause oxidative DNA damage, inhibit the activity of AChE, and increase the levels of IL10 and CRP which place people’s health in danger. Further studies can help identify the types and mechanisms of contaminants in workplaces and the environment, which will help promote individual and social healthcare.
